# Supplementary material for: Social and structural factors associated with substance use within the support network of adults living in precarious housing in a socially marginalized neighborhood of Vancouver, Canada
Source: PLoS One. 2019 Sep 23;14(9):e0222611. doi: 10.1371/journal.pone.0222611 (PMC6756550; doi:10.1371/journal.pone.0222611)
Supplement: S1 Appendix — (PDF) [file pone.0222611.s001.pdf]

S1 Appendix.

Custom-made calculations for age and gender disparity

Age and gender disparity were calculated to compare the age and gender of an ego to all of his or her alters. We coded male as 1, female as 2 and transgender as 3. To calculate the disparity, we subtracted alter gender from ego gender, squared the difference and divided it by the number of ego's supporters (outdegree). Squaring the values was necessary to obtain the absolute distance between genders. Simply calculating the difference could have led to negative and thus misleading results. Values range from 0 to 1 where 0 corresponds to least and 1 to most disparity (i.e. all of an ego's supporters would be of a different gender). In the following formulas, n denotes the number of people in the network.

$$disparity_{gender} = \sum_{i=1}^{n} \frac{(egoGender_i - \ alterGender_i)^2}{egoOutdegree_i};$$

Age disparity was calculated the same way though the difference was not squared since having positive or negative values made a difference in this case. Negative values indicated that the ego was older than his or her supporters and positive values meant that the ego was younger.

$$disparity_{age} = \sum_{i=1}^{n} \frac{(egoAge_i - \ alterAge_i)}{egoOutdegree_i};$$
