# Supplementary material for: Social and structural factors associated with substance use within the support network of adults living in precarious housing in a socially marginalized neighborhood of Vancouver, Canada
Source: PLoS One. 2019 Sep 23;14(9):e0222611. doi: 10.1371/journal.pone.0222611 (PMC6756550; doi:10.1371/journal.pone.0222611)
Supplement: S2 Appendix — (PDF) [file pone.0222611.s002.pdf]

S2 Appendix.

Mixed effect modelling and results

Mixed effect models are used to estimate the relationship between variables. They extend traditional regression models used for cross-sectional data to accommodate longitudinal data. Measurements repeated for an individual will likely be correlated at different time points, violating the independence assumption necessary for a regression. A mixed effect model accounts for these correlations while at the same time estimating the relationship between the outcome variable and the covariates, essentially performing as a modified regression model for longitudinal data (Wu, 2009).

The influence of correlation between repeated measurements for an individual is incorporated through *random effects*. Random effects allow for individual-specific variation over time that can deviate from the population average. They also facilitate accounting for differences between individuals and are estimated from a multivariate normal distribution (Wu, 2009). During the model building process, we allowed for variation over time between repeated measures and for variability within individuals by including random effects for time and individual. In the best fitting models every participant had his or her own intercept but not an individual slope. This means that variation was only assumed for an individual, yet not over time. *Fixed effects* on the other hand, incorporate the population average, i.e. the main effect of an independent variable (Jones, 2017). A specific trajectory with a unique intercept and slope for all is estimated by:

$$Y_{it} = \beta_0 + b_{0i} + (\beta_1 + b_{1i})\, Time_{it} + \beta_2 x_2 + \ldots + \beta_n x_n + \varepsilon_{it},$$

Y_{it} is the binary dependent variable (here personal substance use) for an individual i at time t, and *x* are the independent variables (here alter substance use). In general, β denotes the fixed effects. The parameter β_1 is the fixed effect estimate for time and therefore corresponds to the estimated slope of the population trajectory of the dependent variable. The fixed effect for the independent variables *x* is

denoted by β_n. The random effects of the individuals are accounted for by the b variables, where b_{0i} is the random effect of the intercept and b_{1i} is the random effect of time for an individual i (Jones, 2017). Random errors are accounted for by ε_{it}.

Within the context of substance use dispersion globally on the network, treating alter substance use as an independent variable is an egocentric approximation. We assume that the influence of alter substance use on each ego in the network occurs independently and neglect the influence of second nearest neighbours in the network.

Independence of observations is not a requirement for mixed effect models. They can also accommodate different measurement schedules and an unequal number of assessments per participant (Wu, 2009). Models assume linearity, constant variance, and normal distribution of the residuals which we controlled for during the model building process through visual inspection of the plots. By adding and removing fixed and random effects the models were refined iteratively and the Akaike Information Criterion was used to identify the model with the best fit (Bolker et al., 2009). We explored multiplicative interactions with time using the Wald test, however found additive models to provide the better fit, explaining the total variance by the sum of the covariates. Random intercept models also improved fit. We incorporated up to three predictors in the models, i.e. time, alter substance use, and alter use of substance(s) different from ego use.

The *lme4* package was used to construct logistic mixed effect models and investigate the association between ego substance use (binary dependent variable) and alter substance use (conditional degree, independent variable) (Bates, 2010). The R code is provided in the online repository and the formulas follow the format (random intercept model):

$$Y_{it} \sim Time_{it} + x_2 + \ldots + x_n + (1 \mid Subject),$$

where Y_{it} is personal substance use, x_2 is alter substance use (of the same or other substances) and $(1 \mid Subject)$ are the random effects within an individual.
