# Supplementary material for: Social and structural factors associated with substance use within the support network of adults living in precarious housing in a socially marginalized neighborhood of Vancouver, Canada
Source: PLoS One. 2019 Sep 23;14(9):e0222611. doi: 10.1371/journal.pone.0222611 (PMC6756550; doi:10.1371/journal.pone.0222611)
Supplement: S3 Appendix — (PDF) [file pone.0222611.s003.pdf]

S3 Appendix.

Adapted permutation analysis

An adapted permutation analysis was used to assess whether the association of ego and alter substance use was random in this social network. Keeping the original network structure as well as the respective amount of substance use fixed, the dispersion of personal use was randomized for all seven substances on the entire network 10,000 times. Permutation tests require the randomised samples to be both independent and exchangeable, assumed for our sample (Ge et al., 2018). For each newly generated network with randomised substance use we re-ran the analysis on alter substance use for all egos and using egos only. The mean and standard deviation of these values was compared to the values of the observed data. Small p-values reject the null hypothesis that the association of ego and alter substance use was random in this network. The p-value is the probability for obtaining results at least as large as that observed, provided the null distribution created through randomisation is valid (Knijnenburg et al., 2009). The p-value is calculated as the fraction of randomised values greater than the observed value and the number of permutations performed (N=10,000). We added 1 for both numerator and denominator to avoid understating the p-value (Philipson et al., 2010).

$$\mathrm{p} = \frac{\sum(\text{randomised values} > \text{observed value}) + 1}{\mathrm{N} + 1}\,.$$
