# Supplementary material for: Social and structural factors associated with substance use within the support network of adults living in precarious housing in a socially marginalized neighborhood of Vancouver, Canada
Source: PLoS One. 2019 Sep 23;14(9):e0222611. doi: 10.1371/journal.pone.0222611 (PMC6756550; doi:10.1371/journal.pone.0222611)
Supplement: S4 Appendix — (PDF) [file pone.0222611.s004.pdf]

S4 Appendix.

References for Supplementary Material

(1) Barrera, M., Sandler, I. N., Ramsay, T. B., 1981. Preliminary development of a scale of social support: Studies on college students. American Journal of Community Psychology, 9(4), 435-447.

(2) Bates, D. M., 2010. lme4: Mixed-effects modeling with R. Retrieved from http://lme4.r-forge.r-project.org/book/.

(3) Bolker, B. M., Brooks, M. E., Clark, C. J., Geange, S. W., Poulsen, J. R., Stevens, M. H. H., White, J. S., 2009. Generalized linear mixed models: A practical guide for ecology and evolution. Trends in Ecology and Evolution, 24(3), 127–135. https://doi.org/10.1016/j.tree.2008.10.008

(4) Ge, T., Yeo, BT. T., Winkler, A. M., 2018. A brief overview of permutation testing with examples. Organization for human brain mapping. https://www.ohbmbrainmappingblog.com/blog/a-brief-overview-of-permutation-testing-with-examples [last accessed: 26.10.2018].

(5) Gicas, K. M., Vila-Rodriguez, F., Paquet, K., Barr, A. M., Procyshyn, R. M., Lang, D. J., ... Krajden, M., 2014. Neurocognitive profiles of marginally housed persons with comorbid substance dependence, viral infection, and psychiatric illness. Journal of clinical and experimental neuropsychology, 36(10), 1009-1022.

(6) Irizarry, R., Love, M., n.d., Permutation tests. Biomedical Data Science. http://genomicsclass.github.io/book/pages/permutation_tests.html [last accessed: 26.10.2018].

(7) Jones, A. A., 2018. Longitudinal characterization of psychosis among adults living in marginal housing. University of British Columbia Library, Vancouver.

(8) Linden, I. A., Mar, M. Y., Werker, G. R., Jang, K., Krausz, M., 2013. Research on a vulnerable neighborhood—the Vancouver Downtown Eastside from 2001 to 2011. Journal of Urban Health, 90(3), 559-573.

(9) Phipson, B., Smyth, G.K., 2010. Permutation P-values should never be zero: calculating exact P-values when permutations are randomly drawn. Statistical applications in genetics and molecular biology, 9(1).

(10) Theo A. Knijnenburg, Lodewyk F. A. Wessels, Marcel J. T. Reinders, Ilya Shmulevich., 2009. Fewer permutations, more accurate P-values, Bioinformatics, 25 (12), i161–i168, https://doi.org/10.1093/bioinformatics/btp211.

(11) Wu, L., 2009. Mixed effects models for complex data. Chapman and Hall/CRC.
