# Supplementary material for: Social and structural factors associated with substance use within the support network of adults living in precarious housing in a socially marginalized neighborhood of Vancouver, Canada
Source: PLoS One. 2019 Sep 23;14(9):e0222611. doi: 10.1371/journal.pone.0222611 (PMC6756550; doi:10.1371/journal.pone.0222611)

S1 Fig. Plots of mean alter substance use for all egos where personal substance use was randomised (10,000 iterations, n=201). The x-axis represents the density, the y-axis represents the mean for all alters. The dotted line indicates observed mean alter substance use for all egos.

Methamphetamine (p=0.02)

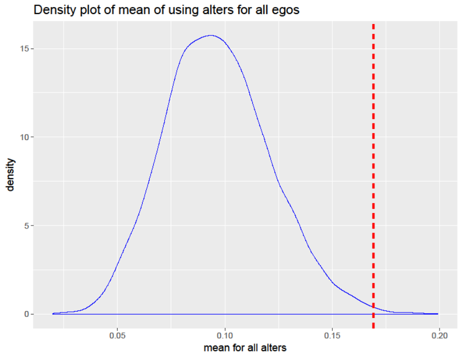

Heroin (p= 0.70)

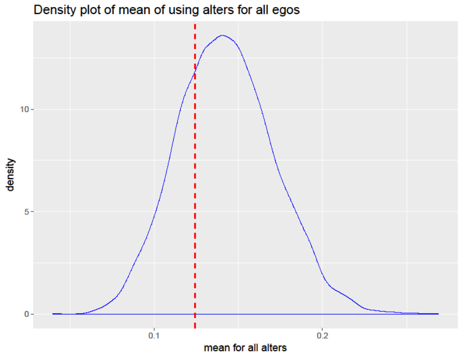

Powder cocaine (p=0.99)

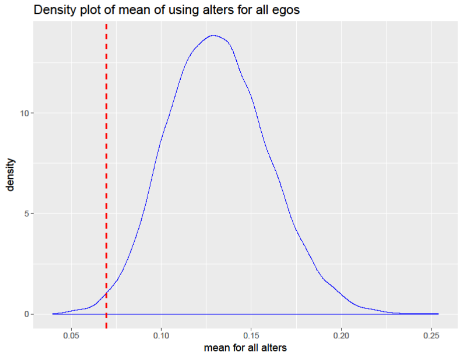

Crack cocaine (p=0.92)

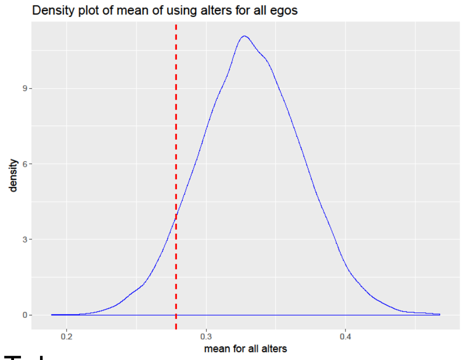

Cannabis (p=0.06)

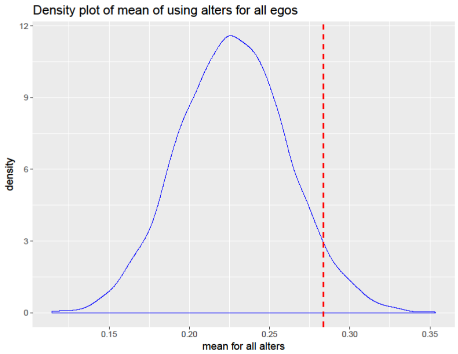

Alcohol (p=0.53)

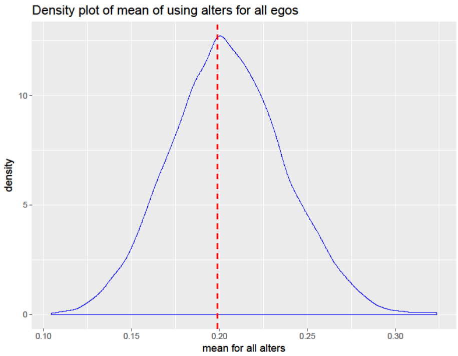

Tobacco (p=0.41)

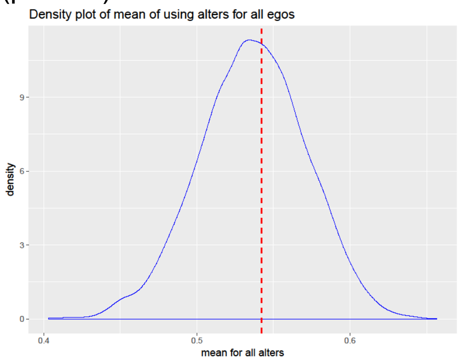

Supplement: S1 Fig — The x- axis represents the density, the y-axis represents the mean for all alters. The dotted line indicates observed mean alter substance use for all egos. (PDF) [file pone.0222611.s005.pdf]
