# Supplementary material for: Social and structural factors associated with substance use within the support network of adults living in precarious housing in a socially marginalized neighborhood of Vancouver, Canada
Source: PLoS One. 2019 Sep 23;14(9):e0222611. doi: 10.1371/journal.pone.0222611 (PMC6756550; doi:10.1371/journal.pone.0222611)

S3 Fig. Plots of mean of alter substance use for egos using the same substance where personal substance use was randomised (10,000 iterations, n=201). The x-axis represents the density, the y-axis represents the mean for all alters. The dotted line indicates observed mean alter substance use for egos using the same substance.

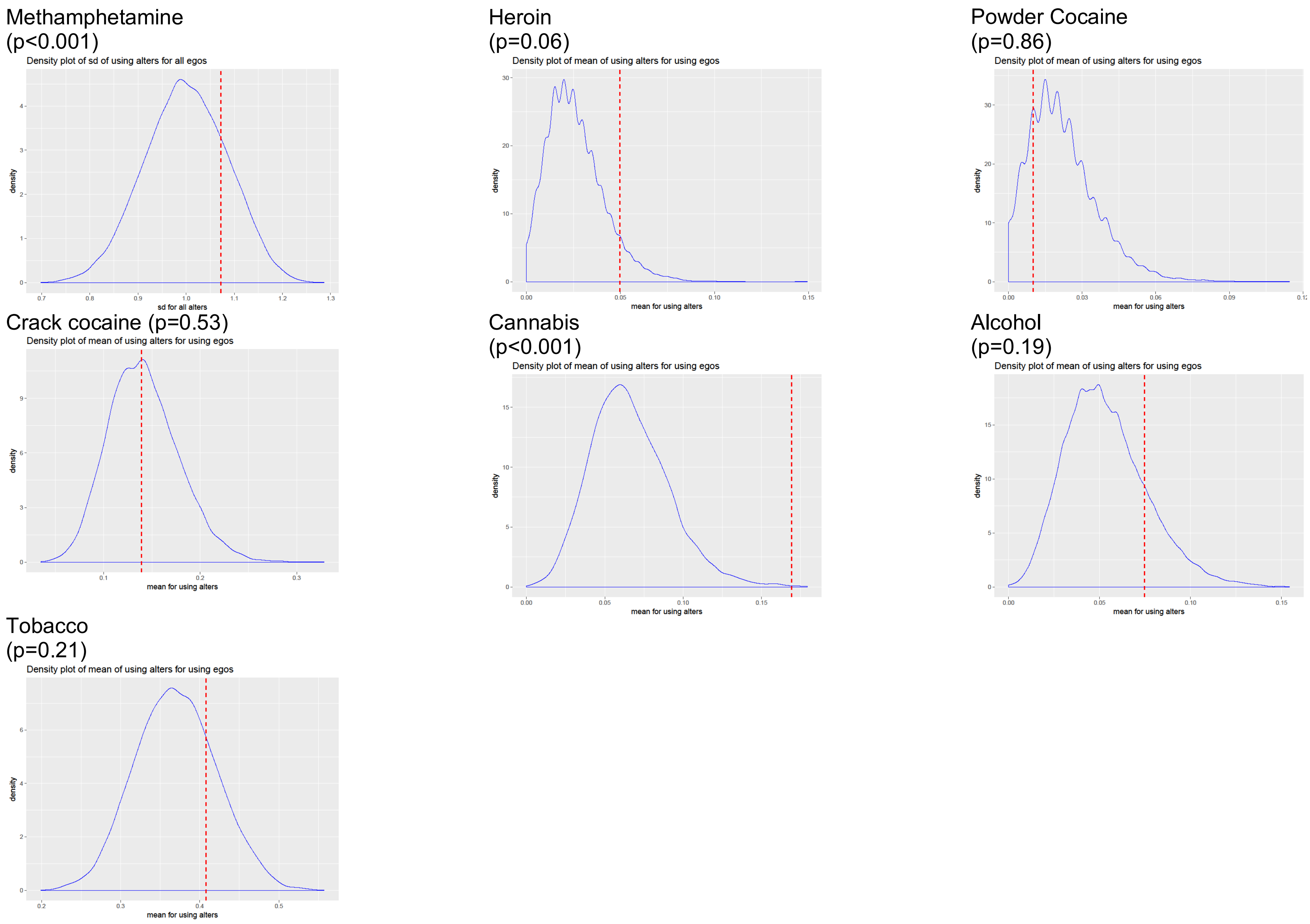

Supplement: S3 Fig — The x-axis represents the density, the y-axis represents the mean for all alters. The dotted line indicates observed mean alter substance use for egos using the same substance. (PDF) [file pone.0222611.s007.pdf]
