# Supplementary material for: Social and structural factors associated with substance use within the support network of adults living in precarious housing in a socially marginalized neighborhood of Vancouver, Canada
Source: PLoS One. 2019 Sep 23;14(9):e0222611. doi: 10.1371/journal.pone.0222611 (PMC6756550; doi:10.1371/journal.pone.0222611)

S4 Fig. Plots of standard deviation of alter substance use for egos using the same substance where personal substance use was randomised (10,000 iterations, n=201). The x-axis represents the density, the y-axis represents the standard deviation for all alters. The dotted line indicates observed standard deviation of alter substance use for egos using the same substance.

Methamphetamine (p=0.001)

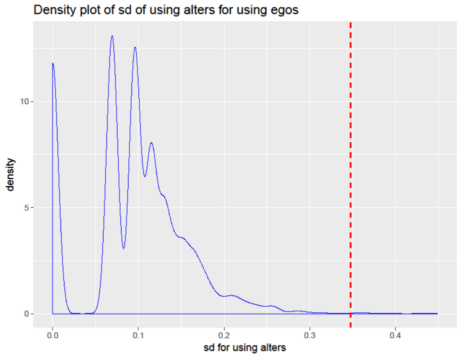

Heroin (p=0.08)

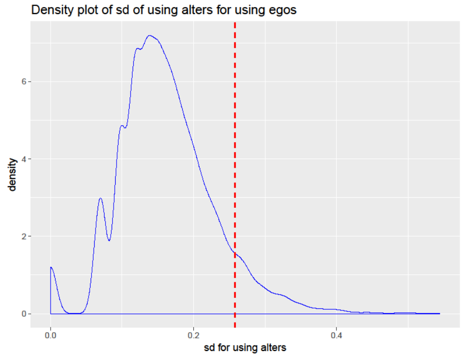

Powder cocaine (p=0.86)

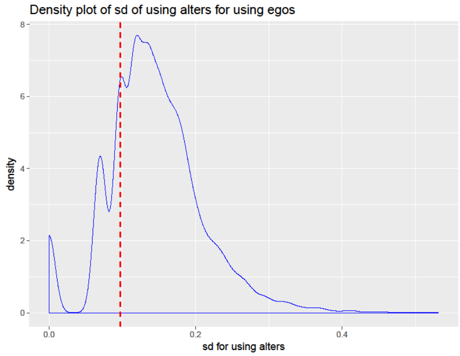

Crack cocaine (p=0.73)

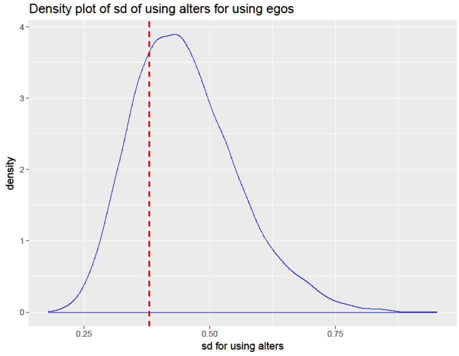

Cannabis (p=0.004)

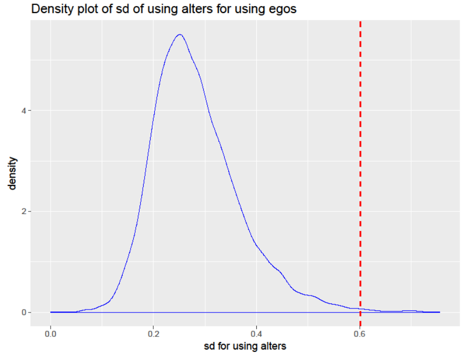

Alcohol (p=0.43)

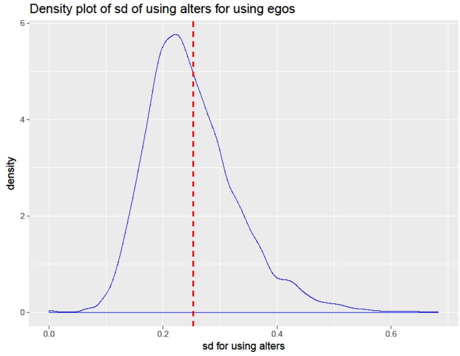

Tobacco (p=0.19)

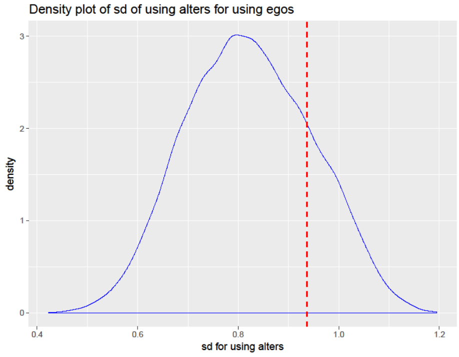

Supplement: S4 Fig — The x-axis represents the density, the y-axis represents the standard deviation for all alters. The dotted line indicates observed standard deviation of alter substance use for egos using the same substance. (PDF) [file pone.0222611.s008.pdf]
