# Supplementary material for: Social and structural factors associated with substance use within the support network of adults living in precarious housing in a socially marginalized neighborhood of Vancouver, Canada
Source: PLoS One. 2019 Sep 23;14(9):e0222611. doi: 10.1371/journal.pone.0222611 (PMC6756550; doi:10.1371/journal.pone.0222611)
Supplement: S1 Table — (PDF) [file pone.0222611.s009.pdf]

**S1 Table.** Demographics and network characteristics of four different graph components.

| | Full network (n = 201) mean (SD) | Cluster1 (n = 37) mean (SD) | Cluster2 (n = 20) mean (SD) | Small components (n = 61) mean (SD) | Isolates (n = 83) mean (SD) |
|---|---|---|---|---|---|
| Age | 44.0 (9.4) | 37.3 (7.8) | 47.7 (6.9) | 46.9 (9.6) | 43.9 (9.1) |
| Male / female / trans (%) | 75 / 25 / 1 | 76 / 24 / 0 | 65 / 35 / 0 | 67 / 33 / 0 | 82 / 17 / 1 |
| Ethnicity: White / Indigenous / Other (%) | 55 / 32 / 12 | 51 / 27 / 21 | 60 / 40 / 0 | 61 / 30 / 10 | 51 / 35 / 14 |
| Education (grade completed) | 10.0 (2.0) | 10.2 (1.5) | 10.2 (2.3) | 9.5 (2.2) | 10.2 (2.1) |
| Time in hotel (months) | 47.1 (44.7) | 53.7 (30.6) | 84.2 (54.2) | 45.9 (45.1) | 36.0 (42.5) |
| Hotel: A / B / C / D / Other (%) | 34 / 33 / 15 / 11 / 6 | 84 / 0 / 0 / 0 / 16 | 0 / 95 / 0 / 0 / 5 | 13 / 30 / 28 / 20 / 10 | 35 / 35 / 17 / 12 / 1 |
| Network descriptive measures | | | | | |
| Age disparity | 0.01 (4.75) | 0.04 (4.75) | 0.21 (5.36) | -0.06 (7.24) | |
| Gender disparity | 0.19 (0.37) | 0.24 (0.36) | 0.32 (0.44) | 0.37 (0.47) | |
| Indegree | 0.78 (1.06) | 1.97 (1.42) | 1.25 (0.91) | 0.95 (0.67) | |
| Outdegree | 0.78 (1.33) | 1.97 (2.19) | 1.25 (1.33) | 0.95 (0.78) | |
| Total degree | 1.55 (2.13) | 3.95 (3.12) | 2.50 (1.61) | 1.90 (1.06) | |
| Positive edges per ego | 0.69 (1.18) | 1.65 (1.99) | 1.20 (1.24) | 0.87 (0.64) | |
| Negative edges per ego | 0.14 (0.49) | 0.38 (0.83) | 0.05 (0.22) | 0.23 (0.56) | |
| Reciprocated edges per ego | 0.36 (0.64) | 0.81 (1.00) | 0.50 (0.61) | 0.52 (0.57) | |
| Betweenness | 3.01 (10.30) | 14.14 (20.48) | 1.90 (3.04) | 0.70 (1.99) | |
| Assortativity degree | 0.36 | 0.14 | -0.20 | 0.10 | |
| Assortativity gender | 0.07 | 0.08 | 0.14 | 0.01 | |
| Density | 0.01 | 0.05 | 0.06 | 0.02 | |
| Alters available | 3.82 (2.70) | 3.22 (2.70) | 4.90 (2.69) | 4.16 (2.67) | |
