# Supplementary material for: Social and structural factors associated with substance use within the support network of adults living in precarious housing in a socially marginalized neighborhood of Vancouver, Canada
Source: PLoS One. 2019 Sep 23;14(9):e0222611. doi: 10.1371/journal.pone.0222611 (PMC6756550; doi:10.1371/journal.pone.0222611)
Supplement: S2 Table — (PDF) [file pone.0222611.s010.pdf]

S2 Table. Substance use in the first month for egos, for all using alters regardless of ego use, and for all using alters for an ego using the same substance.

	MA	Heroin	Cocaine powder	Cocaine crack	Cannabis	Alcohol	Tobacco
Full network (n=201)							
Ego (user / nonuser / NA %)	12 / 62 / 25	18 / 58 / 24	17 / 59 / 24	43 / 33 / 24	29 / 47 / 24	26 / 50 / 24	69 / 7 / 21
Alter (mean, SD)	0.17 (0.62)	0.12 (0.40)	0.07 (0.26)	0.28 (0.56)	0.28 (0.80)	0.20 (0.47)	0.54 (1.07)
Alter with ego user (mean, SD)	0.07 (0.35)	0.05 (0.26)	0.01 (0.10)	0.14 (0.38)	0.17 (0.60)	0.07 (0.25)	0.41 (0.94)
Cluster 1 (n=37)							
Ego (user, nonuser, NA %)	38 / 43 / 19	19 / 62 / 19	3 / 78 / 19	19 / 62 / 19	46 / 35 / 19	14 / 68 / 19	78 / 3 / 19
Alter (mean, SD)	0.86 (1.21)	0.38 (0.72)	0.03 (0.16)	0.24 (0.43)	1.05 (1.47)	0.35 (0.68)	1.54 (1.76)
Alter with ego user (mean, SD)	0.38 (0.71)	0.19 (0.41)	0.00 (0.00)	0.11 (0.22)	0.73 (1.08)	0.11 (0.28)	1.19 (1.54)
Cluster 2 (n=20)							
Ego (user, nonuser, NA %)	0 / 80 / 20	10 / 70 / 20	15 / 65 / 20	70 / 10 / 20	20 / 60 / 20	30 / 50 / 20	70 / 10 / 20
Alter (mean, SD)	0.00	0.15 (0.37)	0.20 (0.41)	0.85 (0.81)	0.25 (0.55)	0.35 (0.59)	0.85 (0.93)
Alter with ego user (mean, SD)	0.00 (0.00)	0.05 (0.16)	0.00 (0.00)	0.55 (0.74)	0.05 (0.20)	0.15 (0.28)	0.55 (0.87)
Small components (n=61)							
Ego (user, nonuser, NA %)	2 / 70 / 28	18 / 56 / 26	18 / 56 / 26	49 / 25 / 26	30 / 44 / 26	36 / 38 / 26	67 / 7 / 26
Alter (mean, SD)	0.03 (0.18)	0.13 (0.34)	0.15 (0.36)	0.49 (0.67)	0.21 (0.49)	0.33 (0.51)	0.57 (0.81)
Alter with ego user (mean, SD)	0.00 (0.00)	0.03 (0.17)	0.03 (0.17)	0.21 (0.40)	0.10 (0.26)	0.13 (0.29)	0.44 (0.72)
Isolates (n=83)							
Ego (user, nonuser, NA %)	12 / 60 / 28	20 / 54 / 25	23 / 52 / 25	42 / 33 / 25	24 / 51 / 25	24 / 51 / 25	66 / 8 / 25

MA: methamphetamine, NA: not available
