# Supplementary material for: Social and structural factors associated with substance use within the support network of adults living in precarious housing in a socially marginalized neighborhood of Vancouver, Canada
Source: PLoS One. 2019 Sep 23;14(9):e0222611. doi: 10.1371/journal.pone.0222611 (PMC6756550; doi:10.1371/journal.pone.0222611)
Supplement: S3 Table — Additive mixed effects modelling results for ego injection substance use (methamphetamine, heroin, powdered cocaine) in month 1 (October 2010) (n = 118). The unadjusted models account for the predictors month i.e. the time of six months and alter injection substance use separately whereas the adjusted models combine these two predictors in one model. (PDF) [file pone.0222611.s011.pdf]

S3 Table. Ego and alter same substance injection use. Additive mixed effects modelling results for ego injection substance use (methamphetamine, heroin, powdered cocaine) in month 1 (October 2010) (n = 118). The unadjusted models account for the predictors month i.e. the time of six months and alter injection substance use separately whereas the adjusted models combine these two predictors in one model.

Factor	Unadjusted Models			Adjusted Models		
	OR	95% CI	p-value	OR	95% CI	p-value
Ego methamphetamine						
Alter methamphetamine	0.52	0.04-7.22	0.68	0.47	0.03-7.07	0.64
Month	1.40	0.99-2.00	0.11	1.41	0.99-2.01	0.11
Ego heroin						
Alter heroin	22.55	5.09-99.93	<0.001	21.84	4.90-97.31	<0.001
Month	0.90	0.73-1.10	0.37	0.95	0.77-1.18	0.70
Ego powder cocaine						
Alter powder cocaine	0.42	0.06-2.93	0.46	0.36	0.05-2.62	0.40
Month	0.91	0.69-1.21	0.60	0.89	0.66-1.19	0.50
