# Supplementary material for: Social and structural factors associated with substance use within the support network of adults living in precarious housing in a socially marginalized neighborhood of Vancouver, Canada
Source: PLoS One. 2019 Sep 23;14(9):e0222611. doi: 10.1371/journal.pone.0222611 (PMC6756550; doi:10.1371/journal.pone.0222611)
Supplement: S4 Table — Additive logistic mixed effects modelling results with three predictors for ego injection substance use (heroin) in month 1 (October 2010) (n = 118). The unadjusted models account for the predictors month i.e. the time of six months, alter injection substance use and another alter injection substance use separately whereas the adjusted models combine these three predictors in one model. (PDF) [file pone.0222611.s012.pdf]

S4 Table. Ego and alter injection use. Additive logistic mixed effects modelling results with three predictors for ego injection substance use (heroin) in month 1 (October 2010) (n = 118). The unadjusted models account for the predictors month i.e. the time of six months, alter injection substance use and another alter injection substance use separately whereas the adjusted models combine these three predictors in one model.

Factor	Unadjusted Models			Adjusted Models		
	OR	95% CI	p-value	OR	95% CI	p-value
Ego heroin						
Alter heroin	22.55	5.09-99.93	<0.001	23.39	4.95-110.59	<0.001
Month	0.90	0.73-1.10	0.37	0.96	0.77-1.18	0.72
Alter methamphetamine	1.99	0.48-8.31	0.43	0.71	0.10-4.85	0.77
Ego heroin						
Alter heroin	22.55	5.09-99.93	<0.001	30.00	5.68-158.55	<0.001
Month	0.90	0.73-1.10	0.37	0.95	0.77-1.18	0.70
Alter powder cocaine	0.89	0.12-6.48	0.93	0.25	0.02-2.52	0.32
