# Supplementary material for: Social and structural factors associated with substance use within the support network of adults living in precarious housing in a socially marginalized neighborhood of Vancouver, Canada
Source: PLoS One. 2019 Sep 23;14(9):e0222611. doi: 10.1371/journal.pone.0222611 (PMC6756550; doi:10.1371/journal.pone.0222611)
Supplement: Attachment — Submitted filename: Response_to_Reviewers.pdf [file pone.0222611.s013.pdf]

August 1, 2019

Dear Prof. Palinkas:

We are submitting revised version of our manuscript on “Social and structural factors associated with substance use within the support network of adults living in marginalized housing in Vancouver” to the PLOS ONE Collection on *Substance Use, Misuse and Dependence: Prevention and Treatment.* We thank both referees for their helpful comments on our original manuscript. As requested, a revised Competing Interests Statement is also included below. I am submitting this revised statement on behalf of all authors.

Response to Reviewer 1:
The introduction has been expanded and a number of references added to better frame the study in the context of previous work on substance use and social network analysis. We have also reviewed previous work on using social network analysis to investigate prevention strategies and improve treatment programs. A conclusion has been added to summarize the implications of our work for prevention, harm reduction, and treatment.

Response to Reviewer 2:
As mentioned above, a conclusion has been added to expand on the implications of our work for using social network analysis to improve prevention, harm reduction, and treatment. Furthermore, the references that were suggested have been added, as well as a number of additional references. We thank the reviewer for drawing this body of work to our attention.

Revised Competing Interests Statement:
I have read the journal's policy and the authors of this manuscript have the following competing interests: AMB has received consulting fees or sat on advisory boards for Bristol-Myers Squibb, Eli Lilly, and Roche. WJP sat on paid advisory boards for Vitality Biopharma, Medipure Pharmaceuticals, and Vinergy Resources; has sat on the board of directors of Abbatis Bioceuticals; and is owner of Translational Life Sciences. WGH received consulting fees or sat on paid advisory boards for: the Canadian Agency for Drugs and Technology in Health, AlphaSights, Guidepoint, In Silico, Translational Life Sciences, Otsuka, Lundbeck, and Newron. This does not alter our adherence to PLOS ONE policies on sharing data and materials.

The manuscript has also been reformatted and I believe that it now conforms to PLOS ONE style requirements.

I am submitting this revised manuscript on behalf of all authors. We confirm that this manuscript has not been published elsewhere and is not under consideration by any other journal. We appreciate your consideration of our revised manuscript and look forward to hearing from you.

Yours sincerely,

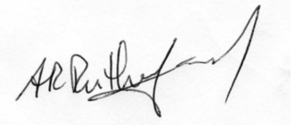

Alexander Rutherford
(on behalf of all authors)
